# Pericarditis and Cardiac Tamponade in Patients Treated with First and Second Generation Bruton Tyrosine Kinase Inhibitors: An Underappreciated Risk

**DOI:** 10.1155/2024/2312182

**Published:** 2024-07-09

**Authors:** Thomas Erblich, Charlotte Manisty, John Gribben

**Affiliations:** ^1^ Centre for Haemato-Oncology Barts Cancer Institute Queen Mary University, Charterhouse Square, London EC1M 6BQ, UK; ^2^ Institute of Cardiovascular Sciences University College London, Gower Street, London WC1E6BT, UK

## Abstract

**Introduction:**

The introduction of Bruton's tyrosine kinase (BTK) inhibitors significantly improved the management of chronic lymphocytic leukemia (CLL). However, BTK carry the risk of cardiotoxicity, which is not only limited to atrial fibrillation. *Case Reports*. We report three cases of patients on BTK inhibitors who developed acute pericarditis and cardiac tamponade. We report the first patient who developed this complication on treatment with zanubrutinib. This patient's treatment was changed to zanubrutinib due to atrial fibrillation. Shortly after cardioversion, he developed cardiac tamponade and shock. He underwent pericardiocentesis, received treatment for acute pericarditis with steroids and colchicine, and made a full recovery. We also report two further cases, both involving patients treated with ibrutinib. These patients also developed acute pericarditis and cardiac tamponade and required pericardiocentesis. All three patients discontinued BTK therapy following the events.

**Conclusions:**

These three cases highlight the rare but potentially life-threatening risk of cardiac tamponade which can occur even with newer generations of BTK inhibitors. Haemato-oncologists should remain vigilant in patients who report dyspnea or who show sinus tachycardia on routine electrocardiography. Even in the absence of classical clinical signs of tamponade, patients require urgent evaluation with echocardiography and potentially emergency pericardiocentesis.

## 1. Introduction

**Figure 1:**
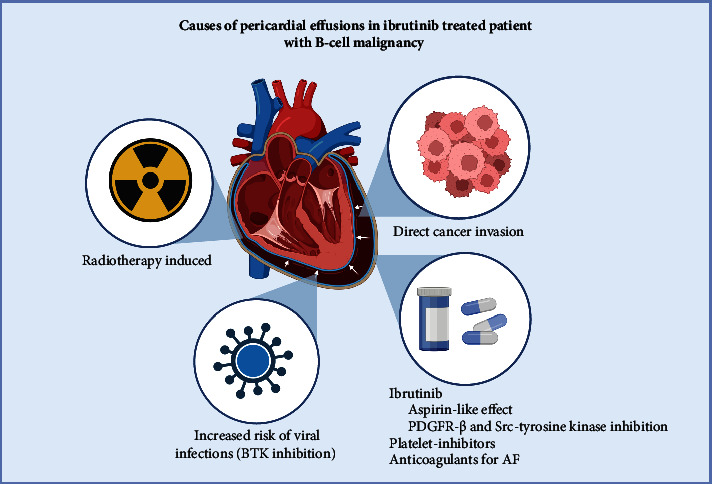
Causes of pericardial effusions in lymphoma patients.

Ibrutinib, an oral once-daily inhibitor of Bruton's tyrosine kinase (BTK), has significantly improved progression-free survival (PFS) and overall survival (OS) in patients with chronic lymphocytic leukemia (CLL) [1]. Its suitability spans a broad patient demographic, encompassing the elderly and those with high-risk disease profiles. The specificity of ibrutinib's action on BTK is limited, resulting in off-target effects in various tissues. Cardiac complications such as atrial fibrillation and hypertension occur frequently [2]. Furthermore, ibrutinib has an “aspirin-like” influence on collagen-induced platelet aggregation and adhesion to von Willebrand factor, which enhances the bleeding risk [3]. There have been instances of cardiac tamponade associated with ibrutinib, especially when used concurrently with antiplatelet therapy [4]. Pericardial effusions in cancer patients can have a multifactorial etiology (Figure 1), attributable to direct neoplastic invasion of the pericardium, hemorrhage, the effects of chemotherapy or radiotherapy, or concurrent infections [5]. Pericarditis and pericardial effusions are infrequently observed in patients treated with ibrutinib [6, 7]. Without prompt and appropriate management, pericarditis can progress to cardiac tamponade [8]. This report presents three cases of pericarditis and cardiac tamponade necessitating hospitalization and decompressive interventions in patients undergoing BTK inhibitor therapy.

## 2. Case 1

**Figure 2:**
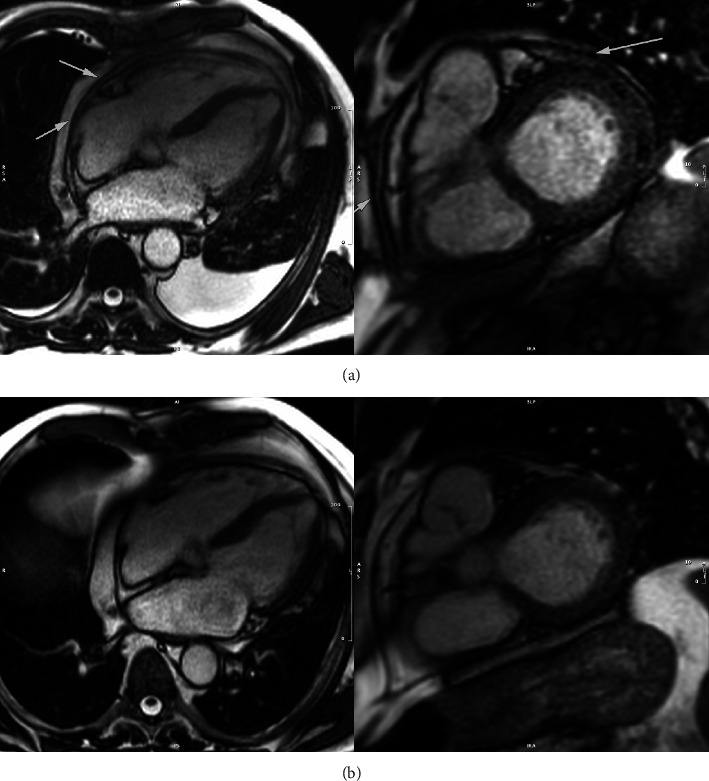
Cardiac MRI images (case 1). (a) Images of postpericardiocentesis showing pericardial thickening with pleural effusion (arrows). (b) Eight weeks later following treatment with steroids and colchicine.

This patient, a 75-year-old male, was diagnosed with chronic lymphocytic leukemia (CLL) five years ago and initially treated with ibrutinib. However, in October 2023, he developed atrial fibrillation. He was anticoagulated and switched to the second-generation TKI zanubrutinib. On December 5th, 2023, he underwent successful DC cardioversion. However, within a week postcardioversion, he experienced increasing shortness of breath and eventually collapsed at home. On admission to the local hospital, he presented in shock with blood pressure of 60/40 mm Hg. A “triple rule out” computed tomography (CT) scan was performed to exclude pulmonary embolus, aortic dissection, and acute coronary event. The scan was negative for all three but revealed a large pericardial effusion. Subsequent echocardiography confirmed a 6 cm effusion with evidence of tamponade physiology with right ventricular diastolic collapse and exaggerated changes in mitral and tricuspid inflow velocities with respiration. He underwent emergency pericardiocentesis with the removal of two liters of serosanguinous fluid with no evidence of malignant cells or infection on fluid analysis. Following drainage, his hemodynamic status improved, and his drain could be removed. Subsequent magnetic resonance imaging (MRI) showed pericardial thickening with evidence of acute pericardial inflammation suggestive of acute pericarditis (Figure 2). The remainder of his cardiac structures and function were within normal limits. Anticoagulation and zanubrutinib were discontinued, and he received a treatment course of steroids and colchicine. Eight weeks later, he remained off BTK inhibitor therapy, and his blood counts continued to be stable. However, he remained breathless. A repeat MRI scan showed resolution of the acute pericarditis, but an ECG confirmed he had reverted to atrial fibrillation with a heart rate of 110 beats per minute. He successfully restarted anticoagulation and is scheduled for repeat DC cardioversion. We are expecting his CLL will eventually progress, in which case we would start venetoclax and obinutuzumab.

## 3. Case 2

**Figure 3:**
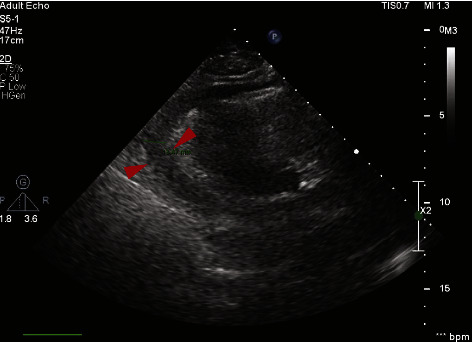
A moderate pericardial effusion with measurements of 1.25 cm adjacent to the right ventricular (RV) free wall, 1.44 cm adjacent to the lateral wall, and 1.24 cm encircling the right atrium (RA). In addition, the left ventricle (LV) displayed diminution, exhibiting midcavity obliteration and generating a late systolic peak gradient of 41 mmHg at rest. Calcification was observed in the mitral and aortic valves, each showing mild stenosis causing some degree of hemodynamic compromise.

An 80-year-old female diagnosed with Waldenström's macroglobulinemia had been under ibrutinib therapy for three years. She had achieved durable disease control with an IgM M-band of <0.5 g/L. She presented with acute breathlessness on minimal exertion and a left swollen and painful leg. Ultrasound Doppler of the leg and a computed tomography pulmonary angiogram (CTPA) were negative for deep vein thrombosis and pulmonary embolism but revealed a large pericardial effusion. An echocardiogram confirmed a moderate circumferential pericardial effusion (Figure 3). Laboratory blood tests showed a CRP of 170 mg/L and impaired renal function with a urea level of 16.5 mmol/L.

She underwent pericardiocentesis where 450 ml of hemorrhagic fluid were drained. Microscopic examination revealed predominantly acute and chronic inflammatory cells, without malignant cells. Flow cytometry confirmed a majority of small, polyclonal B-cells, while T-cells constituted 32% of the total cell, with a CD4/CD8 ratio of 3.59 and natural killer (NK) accounting for 4%. The total protein content was 42 g/L. Both Gram and Ziehl–Neelsen staining failed to demonstrate any organisms. Despite drainage and diuretic treatment, the patient's clinical condition did not improve. Consequently, prednisolone and colchicine were initiated, which resulted in a swift response with a marked decrease in C-reactive protein and a clinical recovery. In view of the cardiac complication, ibrutinib was withdrawn, and she was followed with watchful waiting. Subsequent echocardiograms and a PET-CT scan conducted two months later showed no recurrence of pericarditis. Her IgM levels remained stable for a further six months before rising again. Unfortunately, throughout that period, her overall health had significantly declined; thus, when her IgM levels began rising again, the patient and her family opted for palliative care.

## 4. Case 3

An 87-year-old male, receiving long-term treatment for chronic lymphocytic leukemia (CLL) with ibrutinib developed atrial fibrillation, which was managed with bisoprolol and apixaban alongside ibrutinib. Two weeks before his current presentation, he was admitted to a different hospital due to breathlessness with minimal exertion and pleuritic chest pain. Elevated C-reactive protein (CRP) levels were noted. A chest CT-scan revealed consolidation of the lung and large bilateral pleural effusions. The effusions were drained, and although no organism was confirmed, he received antibiotic treatment with piperacillin/tazobactam and amikacin.

**Figure 4:**
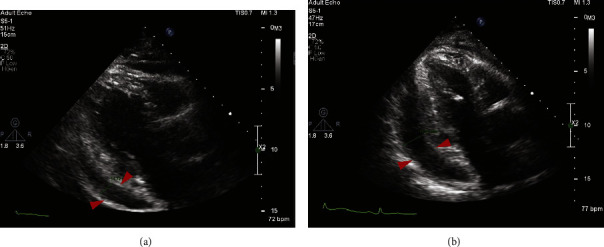
(a) Pericardial effusion measuring 1.5 cm posterior and 1 cm anteriorly not causing hemodynamic consequences. (b) One week later: the pericardial effusion has increased in size and is now causing hemodynamic compromise (case 3).

A week later, he was transferred to our institution. On admission to our hospital, he exhibited a raised jugular venous pressure (JVP) and ankle edema. Although cardiac examination and 12-lead electrocardiograms were normal, echocardiography revealed a mild to moderate circumferential pericardial effusion and reaccumulation of pleural effusions (Figure 4(a)). The pleural effusions were drained, and a second course of antibiotics was administered. The pleural fluid was bloodstained, containing acute and chronic inflammatory cells, with flow cytometry revealing 8% of small cells with a CLL phenotype but no organisms were detected in culture. Respiratory viral PCR testing was incidentally positive for rhinovirus.

His condition initially improved clinically with broad-spectrum antibiotics (piperacillin/tazobactam, amikacin, clarithromycin, and meropenem) but the pericardial effusion persisted. After a week of observation, the effusion had enlarged (Figure 4(b)) and was now hemodynamically compromising. In addition, episodes of rapid atrial fibrillation occurred. Consequently, ibrutinib therapy was discontinued. He underwent a pericardial window procedure during which hemorrhagic fluid was removed. Pericardial biopsies showed no evidence of malignancy.

Postprocedure recovery was uneventful, and he could be discharged from hospital. Ibrutinib was withheld for four months, during which his total white cell count increased to 322. Subsequently, treatment with venetoclax and rituximab was initiated.

## 5. Discussion

To our knowledge, this is the first case report of cardiac tamponade for a patient on zanubrutinib. Patients with cardiac tamponade typically present with hypotension, tachycardia, and jugular venous distension. Detailed clinical examination may reveal muffled heart sounds and *Pulsus paradoxus*. *Pulsus paradoxus* is a misnomer, as it is not strictly paradox but rather exaggerates physiological effects of inspiration [9]. In classic tamponade, patients will exhibit a decrease in stroke volume and blood pressure (>10 mmHg) during inspiration, which results in the “paradox” of audible heart sounds but concurrent loss of a palpable radial pulse. Importantly, patients with cardiac tamponade have reduced renal perfusion and can present with renal failure. Though cardiac tamponade is a clinical diagnosis, echocardiography is important to evaluate the hemodynamic significance of the effusion.

The occurrence of pericarditis and pericardial effusions with ibrutinib has previously been recognized; however, most of the current literature is limited to case reports [4, 7, 10, 11]. A larger meta-analysis of cardiovascular events reported a small risk of pericardial effusions (0.5%) and pericarditis (0.4%) [12]. Thus, the risk of pericardial toxicity appears to be generally low compared to atrial fibrillation; however, it carries a potential risk of life-threatening cardiac tamponade. The problem with pericardial complications in ibrutinib may potentially be wider than generally appreciated. Pharmacovigilance data (VigiBase) contain 81 cases of pericardial effusions, 24 of pericarditis, 16 cardiac tamponades, and 14 pericardial hemorrhages [13]. This could be due to the large number of patients treated with ibrutinib in the hematology practice. Hemorrhagic pericardial effusion combined with pleural effusions has been described by Johnson et al. [10] and is a characteristic of acute pericarditis with pleuropulmonary involvement [8].

The suggested mechanism for BTK to increase the risk of pericardial effusions is inhibition of platelet-derived growth factor receptor *β* (PDGFR-*β*) and Src family tyrosine kinases, which regulate endothelial permeability and interstitial tissue pressure [14]. In addition to serosal inflammation, ibrutinib's “aspirin-like” effect on platelets can aggravate bleeding [4], with major hemorrhage reported in up to 7% of the patients on ibrutinib [1]. The aspirin-like effect of ibrutinib is related to impairment of GPVI-dependent platelet aggregation. Interestingly, this inhibition appears to be limited to thrombus formation around plaques, whilst sparing physiological haemostasias [15]. There is variability in the sensitivity to ibrutinib off-target effects, depending on an individual's drug efflux pump efficacy [16]. The effect is to some extent dose dependent and drugs that block efflux pumps can further increase sensitivity such as calcium channel blockers (e.g., verapamil), which should be avoided when treating atrial fibrillation concomitantly with ibrutinib therapy.

In addition, anticoagulation, which is prescribed for prevention of embolic strokes in atrial fibrillation, can contribute to hemorrhage. However, it is extremely rare for patients on DOACs alone to develop spontaneous cardiac tamponade [17]. Also, DC cardioversion is associated with only a small risk of tamponade (1%) [18]. Together, this suggests that other factors, such as the BTK effects described before potentially in combination with the aspirin-like effect, are required for significant pericardial effusions to develop.

A baseline cardiovascular assessment including evaluation for palpitation, dyspnea, exertional chest pain, edema, unexplained syncope, or arrythmias, together with measurement of pulse and blood pressure, as well as an electrocardiogram and an echocardiogram for patients over 50 is usually obtained before initiating BTK inhibitor treatment [19]. Typically, repeat ECGs are then obtained at periodic clinic visits to screen for new onset atrial fibrillation ECGs. However, the sensitivity of the ECG is low to detect pericardial effusions. In a study by Wang et al. in 216 patients with malignant pericardial effusions, sinus tachycardia was the most common sign (42.1%), whereas more specific signs such as low QRS-voltage or electrical alternans were detected in a minority of patients (17.6% and 21.3%, respectively) [20]. It would be more effective to periodically screen for cardiac symptoms. The most common clinical symptom of tamponade is breathlessness (63%). To confirm tamponade, clinical signs are unreliable. For example, *Pulsus paradoxus* was an insensitive sign only present in 10%. The classical Beck's Triad (hypotension, increased JVP, and quiet/distant heart sounds) detected only a third of the cases. Echocardiography, in contrast, has a sensitivity of 96% and specificity of 98% to detect pericardial fluid, even when performed as a bedside study [21].

The management of acute pericarditis includes high-dose NSAIDs. However, their use in patients concomitantly on ibrutinib can theoretically aggravate the antiplatelet effect. On the balance of risks, we would, therefore, treat with colchicine and steroids instead of NSAIDs. Temporarily discontinuing ibrutinib should be considered where feasible. Following improvement, steroids should be tapered swiftly, while colchicine may be extended for several weeks to ensure full symptom resolution. For those intolerant to ibrutinib, switching to second-generation BTK inhibitors with reduced cardiac cross-reactivity can be considered. Acalabrutinib has demonstrated a lower incidence of adverse cardiac effects compared to ibrutinib [22] and also exhibits less platelet inhibition [16]. Similarly, zanubrutinib in the ASPEN study showed reduced cardiac toxicity [23]; however, as our case report demonstrates, a small risk remains. For high-risk patients who developed cardiac tamponade while on BTK therapy, we would recommend switching to a venetoclax-based regimen. Venetoclax is a BCL2-inhibitor with no direct cardiotoxic effects which has demonstrated high efficacy when combined with obinutuzumab for CLL [24] or as a monotherapy for Waldenström's [25].

## 6. Conclusion

We present the first case report of a patient who develops cardiac tamponade on zanubrutinib. We present two further cases of cardiac tamponade for patients on ibrutinib. BTK can directly and indirectly cause pericarditis and dose-dependent platelet inhibition, which exposes patients to bleeding events. While uncommon, clinicians must be vigilant of the risk of cardiac tamponade, which can develop rapidly and presents a life-threatening complication.

## Data Availability

No additional data were used to support this study.

## References

[1] Barr P. M., Owen C., Robak T. (2022). Up to 8 years follow-up from RESONATE-2: first-line ibrutinib treatment for patients with chronic lymphocytic leukemia. *Blood Advances*.

[2] Sestier M., Hillis C., Fraser G., Leong D. (2021). Bruton’s tyrosine kinase inhibitors and cardiotoxicity: more than just atrial fibrillation. *Current Oncology Reports*.

[3] Hani E., Taha L., Dobson R., Wright D., Lip G. Y. H. (2021). How to manage atrial fibrillation secondary to ibrutinib. *Journal of the American College of Cardiology: CardioOncology*.

[4] Miatech J. L., Hughes J. H., McCarty D. K., Stagg M. P. (2020). Ibrutinib-associated cardiac tamponade with concurrent antiplatelet therapy. *Case Reports in Hematology*.

[5] Chahine J., Shekhar S., Mahalwar G., Imazio M., Collier P., Klein A. (2021). Pericardial involvement in cancer. *The American Journal of Cardiology*.

[6] Styskel B. A., Lopez-Mattei J., Jimenez C. A., Stewart J., Hagemeister F. B., Faiz S. A. (2019). Ibrutinib-associated serositis in mantle cell lymphoma. *American Journal of Respiratory and Critical Care Medicine*.

[7] Aslan Candır B., Yiğenoğlu T. N., Kızıl Çakar M., Dal M. S., Altuntaş F. (2022). Non hemorrhagic pericardial effusion from ibrutinib İn a patient without comorbidities. *Journal of Oncology Pharmacy Practice*.

[8] Bizzi E., Picchi C., Mastrangelo G., Imazio M., Brucato A. (2022). Recent advances in pericarditis. *European Journal of Internal Medicine*.

[9] Swami A., Spodick D. H. (2003). Pulsus paradoxus in cardiac tamponade: a pathophysiologic continuum. *Clinical Cardiology*.

[10] Johnson G., Baviriseaty N., Massanet N., Kooper J. (2023). Serositis causing pericardial and pleural effusions after eight years of maintenance ibrutinib for Waldenstrom’s macroglobulinemia. *Journal of Oncology Pharmacy Practice*.

[11] Kidoguchi K., Kubato Y., Nishimura Y., Kizuka-Sano H., Kimura S. (2021). Ibrutinib-induced cardiac tamponade in chronic lymphocytic leukemia. *Turkish Journal of Haematology*.

[12] Proskuriakova E., Shrestha D. B., Jasaraj R. (2023). Cardiovascular adverse events associated with second-generation Bruton tyrosine kinase inhibitor therapy: a systematic review and meta-analysis. *Clinical Therapeutics*.

[13] Allouchery M., Tomowiak C., Lombard T., Pérault-Pochat M.-C., Salvo F. (2021). Safety profile of ibrutinib: an analysis of the WHO pharmacovigilance database. *Frontiers in Pharmacology*.

[14] Kelly K., Swords R., Mahalingam D., Padmanabhan S., Giles F. J. (2009). Serosal inflammation (pleural and pericardial effusions) related to tyrosine kinase inhibitors. *Targeted Oncology*.

[15] Busygina K., Jamasbi J., Seiler T. (2018). Oral Bruton tyrosine kinase inhibitors selectively block atherosclerotic plaque-triggered thrombus formation in humans. *Blood*.

[16] Series J., Garcia C., Levade M. (2019). Differences and similarities in the effects of ibrutinib and acalabrutinib on platelet functions. *Haematologica*.

[17] Gabarin M., Arnson Y., Neuman Y., Arow Z., Assali A., Pereg D. (2023). Cardiac tamponade in patients treated with direct oral anticoagulants. *The Israel Medical Association Journal*.

[18] Guédon-Moreau L., Gayet J.-L., Galinier M., Frances Y., Lardoux H., Libersa C. (2007). Group of Pharmacology and Therapeutics of French Society of Cardiology, Incidence of early adverse events surrounding direct current cardioversion of persistent atrial fibrillation. A cohort study of practices. *Therapie*.

[19] Quartermaine C., Ghazi S. M., Yasin A. (2023). Cardiovascular toxicities of BTK inhibitors in chronic lymphocytic leukemia. *Journal of the American College of Cardiology: CardioOncology*.

[20] Wang S., Zhao J., Wang C., Zhang N. (2021). Prognosis and role of clinical and imaging features in patients with malignant pericardial effusion: a single-center study in China. *BMC Cardiovascular Disorders*.

[21] Mandavia D. P., Hoffner R. J., Mahaney K., Henderson S. O. (2001). Bedside echocardiography by emergency physicians. *Annals of Emergency Medicine*.

[22] Byrd J. C., Hillmen P., Ghia P. (2021). Acalabrutinib versus ibrutinib in previously treated chronic lymphocytic leukemia: results of the first randomized phase III trial. *Journal of Clinical Oncology*.

[23] Brown J. R., Eichhorst B., Hillmen P. (2022). Zanubrutinib or ibrutinib in relapsed or refractory chronic lymphocytic leukemia. *New England Journal of Medicine*.

[24] Eichhorst B., Niemann C. U., Kater A. P. (2023). GCLLSG, the HOVON and nordic CLL study groups, the SAKK, the Israeli CLL association, and cancer trials Ireland, first-line venetoclax combinations in chronic lymphocytic leukemia. *New England Journal of Medicine*.

[25] Castillo J. J., Allan J. N., Siddiqi T. (2022). Venetoclax in previously treated Waldenström macroglobulinemia. *Journal of Clinical Oncology*.

